# Progesterone protects blood-brain barrier function and improves neurological outcome following traumatic brain injury in rats

**DOI:** 10.3892/etm.2014.1840

**Published:** 2014-07-11

**Authors:** DAOWEN SI, JUAN LI, JIANG LIU, XIAOYIN WANG, ZIFENG WEI, QINGYOU TIAN, HAITAO WANG, GANG LIU

**Affiliations:** 1 School of Basic Medical Sciences, Hebei United University, Tangshan, Hebei 063000, P.R. China; 2 Department of Biochemistry and Molecular Biology, Xinxiang Medical College, Xinxiang, Henan 453000, P.R. China; 3 Department of Neurosurgery, Affiliated Hospital of Hebei United University, Tangshan, Hebei 063000, P.R. China

**Keywords:** progesterone, traumatic brain injury, blood-brain barrier, neurological function

## Abstract

Inflammatory responses are associated with blood-brain barrier (BBB) dysfunction and neurological deficits following traumatic brain injury (TBI). The aim of the present study was to investigate the effects of progesterone on the expression of the inflammatory mediators prostaglandin E2 (PGE2), cyclooxygenase-2 (COX-2), nuclear factor κB (NF-κB) and tumor necrosis factor-α (TNF-α) in the brain, BBB permeability, cerebral edema and neurological outcome, as well as to explore the mechanism of its neuroprotective effect. In this study, male rats were randomly divided into three groups: a sham-operated group (SHAM), a TBI group (TBI) and a progesterone treatment group (TBI-PROG). The TBI model was established using a modified Feeney’s weight-dropping method. Brain samples were extracted 24 h following injury. The expression levels of COX-2 and NF-κB were examined using immunohistochemistry, whilst the expression levels of PGE2 and TNF-α were detected by enzyme-linked immunosorbent assay. BBB permeability was analyzed using Evans blue and cerebral edema was determined using the dry-wet method. The neurological outcome was evaluated using the modified neurological severity score test. The results revealed that progesterone treatment significantly reduced post-injury inflammatory response, brain edema and Evans blue dye extravasation, and improved neurological scores compared with those in the TBI group. In conclusion, the inhibition of inflammation may be an important mechanism by which progesterone protects the BBB and improves neurological outcome.

## Introduction

Traumatic brain injury (TBI) is caused by both primary and secondary injury. Primary injury occurs from the forces at the time of injury and is known to be irreversible. However, it is the complex secondary mechanisms initiated at the time of trauma that have an important role in the delayed progression of the brain damage, which may present novel opportunities for therapeutic strategies. One of the secondary injury processes that may promote delayed neuronal death is post-traumatic inflammation, which has been shown to increase blood-brain barrier (BBB) permeability, cerebral edema and intracranial pressure, resulting in neuronal dysfunction following TBI (1).

During post-traumatic inflammation, metabolic products of arachidonic acid, known as prostanoids, including prostaglandins, prostacyclin and thromboxanes, are released. These aggravate the injury process and have a central role in the central nervous system (CNS) in brain injury (2). Prostaglandin E2 (PGE2) synthesis is regulated by cyclooxygenase (COX) that is present in at least two isoforms, COX-1, the constitutive form, and COX-2, the inducible form (3). COX-2 regulates the critical metabolic step in the biosynthesis of PGE2, which is considered to have a significant role in the inflammatory response following TBI. In addition, nuclear factor κB (NF-κB) is an important transcription factor complex that modulates the expression of numerous genes involved in immune and inflammatory responses, including COX-2 and tumor necrosis factor-α (TNF-α), which are considered to be significant in secondary injury (4). Following TBI, the overproduction of inflammatory mediators within the injured brain, including PGE2, COX-2, NF-κB and TNF-α, are believed to contribute to the cerebral damage, cell death and BBB dysfunction (5). Therefore, inflammation is a significant contributor to secondary injury, and control of the inflammatory response benefits BBB and neurological outcome.

Progesterone is considered to have a neuroprotective effect, and this has been demonstrated in a variety of animal models, including ischemic and traumatic brain insult models (6). Previous studies have shown that post-injury treatment with progesterone decreases brain edema (7), attenuates free radical damage (8), reduces neuronal loss (9), inhibits the inflammatory response (10), restores BBB function (11), increases the levels of endothelial progenitor cells (12) and promotes behavioral recovery (13). In addition, the neuroprotective effect has been confirmed in clinical trials in which progesterone treatment was administered following TBI (14,15). Due to these promising results, a large multicenter phase III clinical trial (ProTECT III) is being conducted in order to further assess the safety and efficacy of this treatment for adults with moderate to severe acute TBI (16). Previous studies have demonstrated that progesterone reduces BBB degeneration (8,11,17–19); however, the mechanism by which progesterone mediates its neuroprotective effects on the BBB has yet to be elucidated. A number of studies have demonstrated that the protective effect of progesterone BBB is associated with reductions of lipid peroxidation and matrix metalloproteinase (MMP) expression (8,11,17–19). Since inflammatory mediators, including PGE2, COX-2, NF-κB and TNF-α, following TBI are associated with the disruption of the BBB, it was hypothesized in the present study that progesterone may promote BBB recovery from TBI by reducing the expression levels of inflammatory mediators. Therefore, the present study investigated whether progesterone inhibits the expression of the important inflammatory molecules PGE2, COX-2, NF-κB and TNF-α following TBI in rats. The ability of progesterone to protect BBB function, reduce brain edema and improve neurological function in the brain-injured rats was also evaluated.

## Materials and methods

### Animals and groups

A total of 90 adult male Sprague-Dawley rats (weighing between 250 and 300 g) were purchased from the Beijing Experimental Animal Center (Beijing, China). The animals were housed in a light controlled room under a 12 h light-dark cycle and maintained at a temperature of 25°C. Animals were given unrestricted access to food and water. All procedures were approved by the Animal Care Committee of Hebei United University (Tangshan, China) and were in accordance with the guidelines of the National Institutes of Health (Bethesda, MD, USA) on the care and use of animals.

The rats were randomly divided into three experimental groups: a sham-operated group (SHAM), a TBI group (TBI) and a progesterone treatment group (TBI-PROG), with 30 rats in each group. Animals in each group were assigned to subgroups for immunohistochemical analysis, enzyme-linked immunosorbent assay (ELISA), detection of Evan’s blue (EB) dye extravasation, determination of brain water content and modified neurological severity score (n=18 per assay; 6 rats from each group).

### TBI model

The TBI model was established on experimental rats by modified Feeney’s weight-dropping method, as previously described (20). In this method, the rats were anesthetized with an intraperitoneal injection of 40 mg/kg sodium pentobarbital. A right parietal craniotomy (5 mm diameter) was drilled under aseptic conditions, 1 mm posteriorly and 2 mm laterally to the bregma. A steel rod (weighing 40 g) with a flat end diameter of 4 mm was allowed to fall from a height of 25 cm onto a piston resting on the exposed intact cranial dura. Rats in the SHAM group were subjected to skull fenestration without brain injury. Rats in the TBI-PROG group received injections of progesterone (Sigma, St Louis, MO, USA), in accordance a previous study (21). Progesterone was dissolved in 22.5% 2-hydroxypropyl-β-cyclodextrin and administered at a dose of 16 mg/kg by intraperitoneal injection 1 h post injury to ensure more rapid absorption following TBI. Subsequent injections of 16 mg/kg were administered subcutaneously 6 h and 12 h following TBI. Rats in the SHAM and TBI groups received equivalent volumes of 2-hydroxypropyl-β-cyclodextrin at the same time following the surgery.

### Immunohistochemistry

The animals were decapitated 24 h following injury for tissue assays. The rats were anesthetized and perfused through the heart with 4% paraformaldehyde. Brains were carefully removed and immersed in fixative. A 3 mm-thick coronal slice of each brain, containing the area surrounding the injury site, was dehydrated and embedded in paraffin. Coronal sections of the brain (10 μm thickness) were cut and rinsed in phosphate-buffered saline (PBS). The sections were incubated in PBS-Triton (PBS-T) containing 0.1% H_2_O_2_ for 30 min to block the endogenous peroxidase. The sections were rinsed three times for 5 min in PBS-T and incubated with 1.5% normal goat serum for 30 min. The sections were then incubated overnight (4°C) using anti-COX-2 (Santa Cruz Biotechnology, Santa Cruz, CA, USA) or anti-NF-κB (Santa Cruz Biotechnology) primary antibodies, diluted 1:100 with PBS. Following extensive rinsing steps in PBS, sections were reincubated in biotinylated goat anti-rabbit antibody (Zhongshan Biotechnology Co., Ltd., Beijing, China) for 1 h at room temperature. The immunocomplex was visualized using diaminobenzidine as a chromogen in a reaction with peroxidase. For negative controls, the primary antibody was omitted.

### Enzyme-linked immunosorbent assay (ELISA)

The animals were decapitated 24 h after brain injury. The brain tissues were isolated from the skull and washed with ice-cold physiological saline to remove surface blood. The cortex was then separated, weighed and placed in a homogenizer. The tissue was homogenized with 1 ml ice-cold physiological saline per 100 mg brain tissue. Hypothermal centrifugation was performed at 302 × g for 10 min, and the supernatant was obtained. An ELISA kit (Shanghai Saimo Biotechnology, Shanghai, China) on a microplate reader (Hyperion MR III; Hyperion Inc., Miami, FL, USA) was used to determine the levels of PGE2 and TNF-α.

### Detection of EB dye extravasation

The BBB permeability was determined by EB extravasation 24 h following TBI. A dose of 2 ml/kg 2% EB was injected intravenously through the tail vein. Animals were anesthetized after 1 h and perfused using saline to remove intravascular EB dye. Animals were then decapitated, the brains removed and homogenized in PBS. The protein was precipitated by adding trichloroacetic acid, and the samples were cooled and centrifuged. The resulting supernatant was measured for absorbance of EB at 610 nm using a spectrophotometer (model VIS722; Aoxi, Shanghai, China).

### Determination of brain water content

Coronal sections of the frontal cortex of the brain (4 mm thick) were weighed to obtain the wet weight, placed in an oven at 90°C for 2 days and then weighed again to obtain the dry weight. The formula used to calculate the water content was as follows: Brain water (%) = [(wet weight-dry weight)/wet weight] × 100.

### Modified neurological severity score (mNSS)

The neurological function of the rats was assessed using the mNSS test (22), which includes motor, sensory, reflex and balance tests. In the mNSS test, the neurological function was graded on a scale of 0–18. The test was performed 24 h following TBI in a blinded manner. The higher the score, the more severe the injury. A score of 0 indicates no neurological deficit, whilst a score of 18 indicates the most severe impairment.

### Statistical analysis

All results are expressed as mean ± standard error of the mean. SPSS software, version 16.0 was used for statistical analysis of the data (SPSS, Inc., Chicago, IL, USA). A one-way analysis of variance was performed to determine the differences among the groups followed by a least significance difference in order to compare the differences between groups. P<0.05 was considered to indicate a statistically significant result.

## Results

### Progesterone treatment inhibits COX-2 and NF-κB expression

**Figure 1:**
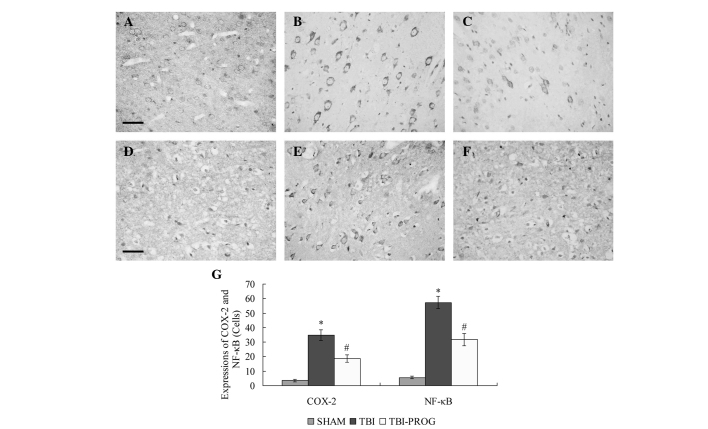
Immunohistochemical analysis of COX-2 and NF-κB in the cortex of rats in the SHAM, TBI and TBI-PROG groups. Immunohistochemistry of (A–C) COX-2 and (D–F) NF-κB expression. (A, D) The SHAM group showed few positive cells, (B,E) The TBI group showed strongly stained positive cells, and (C, F) the TBI-PROG group showed fewer positive cells compared with the TBI group (scale bar, 50 μm; magnification, ×400). (G) Administration of progesterone significantly inhibited the TBI-induced upregulation of COX-2 and NF-κB expression in the cortex (n=6/group); ^*^P<0.05, compared with the SHAM group; ^#^P<0.05, compared with the TBI group. COX-2, cyclooxygenase 2; NF-κB, nuclear factor κB; TBI, traumatic brain injury, PROG, progesterone.

Immunohistochemical analysis revealed that the expression levels of COX-2 and NF-κB in the cortex were low in the SHAM group. Following TBI, significant increases in COX-2 and NF-κB expression levels were observed in the cortex of rats in the TBI group compared with those in the SHAM group (P<0.05). The expression levels of COX-2 and NF-κB were decreased by progesterone administration in the TBI-PROG group compared with those in the TBI group (P<0.05; Fig. 1).

### Progesterone treatment decreases PGE2 and TNF-α levels

The expression levels of PGE2 and TNF-α were low in the brains of rats in the SHAM group. Compared with those in the SHAM group, the PGE2 and TNF-α levels were markedly increased following TBI (P<0.05), whilst the administration of progesterone significantly decreased the PGE2 and TNF-α expression levels compared with those in the TBI group (P<0.05; Table I).

### Progesterone treatment decreases BBB permeability

Low EB extravasation was observed in rats in the SHAM group. However, rats in the TBI group demonstrated a significant increase in BBB permeability compared with that of the rats in the SHAM group (P<0.05). Progesterone significantly inhibited EB extravasation in the animals with brain injury (P<0.05; Table I).

### Progesterone treatment reduces brain water content

The brain water content in the TBI group was significantly increased compared with that in the SHAM group (P<0.05). Following progesterone administration, the brain water content in the TBI-PROG group was significantly reduced compared with that in the TBI group (P<0.05; Table I).

### Progesterone treatment improves neurological outcome

The mNSSs for rats in the SHAM group were low (0.5±0.54). The mNSSs for rats in the TBI-PROG group (10.5±1.37) were significantly lower following TBI compared with those of rats in the TBI group (14.8±2.75; P<0.05). These results indicate that progesterone treatment improves the neurological functional outcome following TBI in rats.

## Discussion

In the present study, it was demonstrated that TBI results in an increased expression of PGE2, COX-2, NF-κB and TNF-α in the cortex in rats, and the increase in these inflammatory mediators in the brain following TBI was associated with BBB dysfunction, brain edema and the development of functional deficits. Progesterone was observed to significantly reduce post-injury inflammatory response, BBB permeability and cerebral edema, as well as improve functional outcome.

Inflammatory processes are considered major components of the secondary injury cascade following TBI (23). PGE2, the metabolic product of arachidonic acid, is a key regulator. Overexpression of PGE2 produced by COX-2 is an important determinant of the cytotoxicity associated with inflammation following injury to the brain (24). PGE2 may affect pathological processes through the modulation of glutamate release, cerebral vasoconstriction and neuroendocrine function (24). Furthermore, PGE2 has been shown to be associated with the generation of highly reactive oxygen species (ROS), which have potential deleterious effects on lipids, proteins and DNA, and lead to a breakdown of the BBB following TBI (25). In addition, NF-κB is an upstream regulator of inflammation that activates TBI-induced inflammatory molecules, including COX-2 and TNF-α (26). TNF-α is involved in BBB injury and edema formation through a mechanism involving MMP upregulation (11). In combination, this indicates that secondary inflammation exacerbates BBB dysfunction following TBI.

Progesterone has been shown to be a potent antagonist of CNS inflammation and cerebral edema following TBI (6). Previous studies in aged rats showed that progesterone decreases acute inflammation by preventing the expression of inflammatory mediators, including COX-2, IL-6 and NF-κB following TBI (27). Pettus *et al* (28) suggested that progesterone administered following TBI may reduce the initial cytotoxic surge of inflammatory factors, including complement factor C3, glial fibrillary acidic protein and NF-κB. Progesterone may also reduce the injury-induced expression of inflammatory mediators interleukin-1 β (IL-1β) and TNF-α (29). The fact that progesterone reduces inflammation following TBI may also contribute to the widely observed beneficial effects of the hormone on edema (28). Administration of progesterone following brain injury has been shown to attenuate edema, even when the progesterone treatment was delayed for up to 24 h following injury (30). In the present study, it was found that progesterone treatment inhibited the expression of the inflammatory mediators PGE2, COX-2, NF-κB and TNF-α that accompanied TBI, and it was observed that brain edema in the injured rat brain following TBI was decreased by progesterone administration, which is in agreement with previous studies (27–29).

The protective effect of progesterone on the BBB has been previously investigated and a number of studies have suggested that treatment with progesterone following TBI attenuates BBB permeability following brain injury (8,11,17–19). Ishrat *et al* (19) studied the changes of MMPs and inflammation following permanent middle cerebral artery occlusion (pMCAO). The authors found that ischemic injury significantly increased the expression levels of MMP-9, MMP-2, TNF-α and IL-6, and EB extravasation, but progesterone attenuated BBB disruption by reducing MMP levels and the inflammatory response. A previous study has also shown BBB leakage to be significantly decreased in progesterone-treated animals following TBI, and this decrease was accompanied by a reduction in lipid peroxidation (8). Although previous studies have demonstrated that the protective effect of progesterone on BBB may be associated with a reduction of lipid peroxidation, reduction of MMP expression and attenuation of TNF-α and IL-6 levels, little is known about the effect of progesterone on BBB permeability and the expression levels inflammatory mediators COX-2, PGE2 and NF-κB in the brain. In the present study, it was demonstrated that TBI in rats results in reductions in the expression levels of PGE2, COX-2, NF-κB and TNF-α in the cortex, as well as BBB dysfunction, and that the administration of progesterone significantly reduces the expression levels of these inflammatory mediators. Inflammation is known to contribute to secondary BBB damage, which may explain the mechanism of the effect of progesterone on the BBB. In addition, in the present study it was also demonstrated using the mNSS test, which is a well-established scoring system to determine the neurologic performance of rats following brain injury (22), that the administration of progesterone is able to enhance functional recovery. Since BBB disruption is considered to be associated with the development of neurological deficits following TBI (31), the improvement of neurological function by progesterone may be linked with the protection of BBB function.

In conclusion, the present study demonstrated that progesterone treatment is able to significantly inhibit the inflammatory response, attenuate BBB disruption and brain edema and improve functional recovery following traumatic brain injury. Since the inflammatory response is a cause of considerable secondary BBB damage following brain trauma, the inhibition of inflammation may be another crucial mechanism by which progesterone protects the BBB and improves neurological outcome.

## Figures and Tables

**Table I:** Effects of PROG on PGE2, TNF-α, blood-brain barrier permeability and WC in the cerebral cortex following TBI in rats.

Group	PGE2 (ng/g)	TNF-α (ng/g)	EB (μg/g)	WC (%)
SHAM	2.3±0.51	0.6±0.18	3.2±0.54	74.3±0.92
TBI	7.1±0.94^a^	1.7±0.32^a^	14.6±1.79^a^	82.6±2.13^a^
PROG	4.5±0.68^b^	1.2±0.29^b^	8.3±1.87^b^	78.4±1.47^b^

Data are presented as the mean ± standard error of the mean (n=6).

a P<0.05 vs. SHAM group;

b P<0.05 vs. TBI and SHAM groups.

PGE2, prostaglandin E2; TNF-α, tumor necrosis factor α; EB, Evans blue; WC, water content; TBI, traumatic brain injury, PROG, progesterone.
